# Proactive Personality Measurement Using Item Response Theory and Social Media Text Mining

**DOI:** 10.3389/fpsyg.2021.705005

**Published:** 2021-07-22

**Authors:** Gancheng Zhu, Yuci Zhou, Fengfeng Zhou, Min Wu, Xiangping Zhan, Yingdong Si, Peng Wang, Jun Wang

**Affiliations:** ^1^Key Laboratory of Symbolic Computation and Knowledge Engineering of Ministry of Education, College of Computer Science and Technology, Jilin University, Changchun, China; ^2^Normal College, Weifang Institute of Technology, Weifang, China; ^3^School of Psychology, Shandong Normal University, Jinan, China

**Keywords:** measurement, proactive personality, item response theory, text mining, machine learning

## Abstract

This prospective study was designed to propose a novel method of assessing proactive personality by combining text mining technology and Item Response Theory (IRT) to measure proactive personality more efficiently. We got freely expressed texts (essay question text dataset and social media text dataset) and item response data on the topic of proactive personality from 901 college students. To enhance validity and reliability, three different approaches were employed in the study. In Method 1, we used item response data to develop a proactive personality evaluation model based on IRT. In Method 2, we used freely expressed texts to develop a proactive personality evaluation model based on text mining. In Method 3, we utilized the text mining results as the prior information for the IRT estimation and built a proactive personality evaluation model combining text mining and IRT. Finally, we evaluated those three approaches *via* the confusion matrix indicators. The major result revealed that (1) the combined method based on essay question text, micro-blog text with pre-estimated IRT parameters performed the highest accuracy of 0.849; (2) the combined method using essay question text and pre-estimated IRT parameters performed the highest sensitivity of 0.821; (3) the text classification method based on essay question text had the best performance on the specificity of 0.959; and (4) if the models were considered comprehensively, the combined method using essay question text, micro-blog text, and pre-estimated IRT parameters achieved the best performance. Thus, we concluded that the novel combined method was significantly better than the other two traditional methods based on IRT and text mining.

## Introduction

“How is career success achieved?” This question has aroused extensive thinking and discussion, and numerous psychologists have explored this topic from different perspectives and produced an abundance of research results (Sutin et al., 2009; Converse et al., 2012). Those previous results emphasized the relationship between personalities and individual success; among these studies, proactive personality is believed to be one of the key factors that affects individual success (Seibert et al., 1999, 2006; Thompson, 2005). People with this trait have the ability to consciously select and influence their surroundings for resources that contribute to the success (Prieto, 2010; Neneh, 2019). Additionally, Pan et al. (2018) demonstrated that career adaptability mediated the positive relationship between proactive personality and employment success.

In the measurement of proactive personality, most researchers have relied on the traditional self-report questionnaire. Even without motivation to fake, self-report could be distorted by self-presentation (Hogan et al., 1996; Hickman et al., in press). However, personality prediction models developed with text mining technology showed great potential in replacing the traditional approach (Azucar et al., 2018). Nowadays, social media platform user generates abundant text data and the technology of text mining develops more and more rapidly, which brings new opportunities for researchers to measure proactive personality. Previous studies have confirmed that text data can help improve the accuracy of clinical test scores (He, 2013). Thus, the target of this study was to combine freely expressed text data (essay question text and micro-blog) with questionnaire data, in order to find out the optimal approach to measure proactive personality.

## Related Work

### Proactive Personality

Bateman and Crant (1993) introduced the concept of proactive personality in organizational behavior research and believed that proactive personality is a positive trait that drives individuals to take actions to avoid being restricted by situational factors. According to Campbell (2000), a proactive personality contains five core characteristics: high job competence; interpersonal competence, leadership, and trustworthiness; a high level of organizational commitment and responsibility; proactive qualities such as initiative and independent judgment; and the quality of integrity and a higher-value pursuit.

The research of proactive personality has been associated with career development. Fu and Rebecca (2016) found that proactive personality positively predicted career achievements. Seibert et al. (1999) analyzed the differences between proactive individuals and non-proactive individuals and found that proactive individuals are more active in career planning and work arrangements in daily work. Parker et al. (2010) showed that employees with proactive personalities have better performance than employees without this trait do. Proactive personality plays a very positive role in stimulating internal motivation (Chang et al., 2014); therefore, individuals with proactive personalities tend to influence their environment actively and to seek new methods to improve their performance (Zhang and Yang, 2017). Studies also found that proactive personality is related to the innovative behaviors of teachers (Kong and Li, 2018). Additionally, it is reported that proactive personality is positively related to the career planning of graduates (Valls et al., 2020) and employment behaviors of college students (Claes and Witte, 2002); proactive personality has a significant impact on the entrepreneurial intentions of university students (Mustafa et al., 2016). For career decision-making, it is proved that the career decision-making difficulties of college students are negatively predicted by proactive personality (He et al., 2020). Xin et al. (2020) suggested that a strong proactive personality plays a prominent role in career success criteria, and individuals with strong proactive personalities have more confidence in making career decisions.

### Item Response Theory

Classical Test Theory (CTT) has the advantage of convenience and standard uniformity, but it has inevitable disadvantages such as the dependence of specific tests, the dependence of test parameter estimation of the sample, the inaccuracy of test error estimation, and the limitations of measurement results promotion (Fan, 1998; Dai and Luo, 2013). Item Response Theory (IRT) overcomes some limitations of CTT and lies in the assumption of no relationship between all the statistical indexes and samples.

Psychometricians are committed to exploring its structure and properties from the perspective of measurement and quantify it to measure the quantity (or status) of individuals with these traits, to further predict individual behavior (Dai and Luo, 2013; Reise and Rodriguez, 2016). Generally, in psychological measurement, there is no linear relationship between the reaction of subjects and the latent trait on a certain item. Item Response Theory updates and optimizes the algorithm on this basis, which could perform a more accurate analysis and estimation of the non-linear model and better meet the needs of modern analysis.

Item Response Theory is an advanced theory of psychological and educational measurement, which is used to study the relationship between response behavior and latent traits. The parameter estimation of the IRT model is independent of samples, namely, the estimation of ability parameter of subjects does not depend on the difficulty of items (Dai, 2015); therefore, even if the measurement scale is different, they can be compared directly. Item Response Theory has many potential advantages over CTT. First, the IRT model can obtain stable estimations of item parameters, latent traits, standard errors on trait-level conditions, and traits based on item content. Second, IRT also helps evaluate the different item functions, including the different response modes or different people on the same scale, and further to implement the computerized adaptive testing. Item Response Theory can also help develop improved proactive personality evaluation indicators (Hays et al., 2000).

There are many basic models of IRT: Normal Ogive Model, Rasch model, and logistic model (Luo, 2012). Generally, Graded Response Model (GRM) was applied to intermittent scales, such as Likert-type scale (Yaar, 2019). Because our experimental materials include Likert-type scale data, the model applied in this study is GRM.

The GRM was proposed by Samejima (1969). It applies to Likert-scale data (the scores obtained by selecting different options are ranged from 0, 1,. *m*), rating scale data (such as the student's grade is rated as excellent, good, medium, or poor), etc. Samejima assumes that each response category has a characteristic curve. If the participants' response to item *i* is divided into *m* + *1* categories, their scores are *x* = *0* to *m*, if *P*_*i*_*x*__(θ) is the probability that the subject with the ability is scored *i*_*x*_, $P_{i_{x}}^{*}\left(\theta \right)$ is the probability that the subject with the ability is scored greater than or equal to *i*_*x*_. The two-parameter logistic model in this paper is:

(1) $$\begin{array}{l} \begin{array}{l} P_{i_{x}}^{*}\left(\theta \right)=\;\frac{1}{1+e^{-\;D{\;}^{*}a_{i}\left(\theta \;-\;b_{i_{x}}\right)}} \end{array} \end{array}$$

where *a*_*i*_ denotes the discrimination of the *i*-th item, *D* = 1.702 in general is a constant, θ represents the latent trait, and *b*_*i*_*x*__ is the grade difficulty of the *x*-th level of the *i*-th item. We define $P_{i_{0}}^{*}=\;P^{*}\left(u=0|\theta \right)=1$ and $P_{i_{m}}^{*}=\;P^{*}\left(u=m+1|\theta \right)=1$, where *u* represents subject's selecting the *m*+*1*-th category of the *i*-th item. As a result, the probability that the participant answers category *x* correctly is:

(2) $$\begin{array}{l} \begin{array}{l} P_{i_{x}}\left(\theta \right)=P_{i_{x}}^{*}-P_{i_{x+1}}^{*}\; \end{array} \end{array}$$

Using item response model to analyze different parameters is the basic and critical step, and the response matrix can play an important role in this process. According to the response of the subjects to the items, the parameters of their abilities and the items can be deduced. The IRT parameter estimation methods mainly include maximum-likelihood estimation, the EM algorithm, Bayesian estimation, and Bayesian expectation-maximization-maximization based on the Metropolise-Hastings Robbins-Monro algorithm (BEMM-MH-RM) (Cai, 2008; Guo, 2018). This paper mainly introduces Bayesian estimation and its two extended algorithms.

Cai (2008, 2010a,b) combined some principles and methods of Markov chain Monte Carlo, Robbins-Monro, and the EM algorithm to the purpose the Metropolis-Hastings algorithm. Compared with the traditional Bayesian algorithm, the new algorithm uses parallel sampling to collect data and then uses the integration method to analyze data. To simultaneously ensure the accuracy of output, the new algorithm also introduces the parameters of the ability of subjects. Based on the calculation of expectation variables, this algorithm realizes the split of the likelihood function and narrows the scope of estimates. Additionally, two maximization steps continuously iterate the obtained results; thus, the stability of estimated results is further improved (Guo, 2018). In general, MH-RM has two major steps in estimation. The first step is mainly to conduct a higher-dimensional integral calculation, and the second step is to perform an iterative analysis of the relevant parameters. Cai (2008) argued that this method is especially suitable for functions with missing parameters. Compared with Newton–Raphson iteration, the MH-RM method can better improve the accuracy and stability of estimation results. Therefore, the MH-RM was employed as the estimation method in this study.

### Text Mining and Personality Assessment

With the rapid development of big data analysis, more and more social media text extracted from platforms (e.g., Facebook, Twitter, YouTube, Sina Weibo, etc.) was applied in the field of psychology research (Woo et al., 2020). A number of researchers used text mining technology to design predictors for the recognition of personality traits. For example, Hassanein et al. (2018) proposed a method for personality traits prediction based on text semantic analysis. Souri et al. (2018) represented a model based on boosting-decision tree to recognize user personality by their profile on Facebook. Sarwani et al. (2019) using Facebook status document developed the approach to predict The Big Five personality. In addition to developing prediction tools, machine learning and text mining techniques were also applied to the field of personality assessment.

Machine learning provides an unprecedented opportunity for the development of personality assessment and theory (Bleidorn and Hopwood, 2019). Personality assessment based on machine learning can overcome the shortage of traditional personality testing based on CTT or IRT and aim at understanding human personality and developing personality theory (Ock and An, 2021). Cooper et al. (2020) explored to assess personality *via* the situational and behavioral features of Instagram photos; results indicated that personality is related to the contextual cues, characteristics, categories, behaviors, and emotions of Instagram photos. Similarly, social media text mining technology was also applied in personality assessment such as the work of Ahmad and Siddique (2017) and the work of Adamopoulos et al. (2018). Additionally, the texts of an individual diary or interview are good materials to extract individual psychological characteristics. The work of Zimmermann et al. (2019) concluded that, by applying measures of a personality dynamics diary, clinicians may deeply understand the causes and maintenance of maladaptive dispositions of a person and ultimately find personalized leverage point for treatment intervention, though this process may require a lot of manpower to collect research texts and to perform qualitative analysis. Text mining can help to understand complex written analytical systems (Rzhetsky et al., 2009; Al-Daihani and Abrahams, 2016) and has been widely used in related research. For instance, Zhu et al. (2015) believed that the psychological characteristics of individuals could be revealed by their social network contents; it is possible to analyze personal psychological characteristics, mental health, social attitudes, and other information through the text mining of Weibo data (Li et al., 2015; Liu and Zhu, 2016; Zhou et al., 2017; Yilmaz et al., 2019; Hutama et al., 2020).

### This Study

This paper proposed a novel method that combined text mining with IRT to process structured and unstructured data in a system framework. The individual analysis process of proactive personality includes two main stages, namely, text classification training and evaluation, and the estimation of proactive personality. The Bayesian method is suitable for this type of hierarchical estimation. We used the combined method based on text analysis and IRT model in the Bayesian framework, in which the text classification scores from essay questions and the micro-blog texts of each subject are set as prior data. Then, we used IRT model to estimate the ability parameters of samples. The formula obtained is:

(3) $$\begin{array}{l} \begin{array}{l} P\left(\theta |x,y\right)\propto P\left(x|\theta ,\alpha ,\beta \right)g\left(\theta |y\right)\; \end{array} \end{array}$$

Based on the analysis of formula (3), *y* denotes the score of an individual; *g*(θ|*y*) represents the analysis model based on the individual score; α and β, respectively, denote the discrimination parameter and the difficulty parameter; and *P*(*x*|θ, α, β) represents the likelihood function of the algorithm (He, 2013; Zhang et al., 2019).

This study combines subjective and objective information; namely, we use text mining and IRT as subjective measurement and objective measurement of proactive personality. Additionally, for the subjective measurement, we collected the self-reported text of subjects (proactive personality essay questions of college students) and obtained their micro-blog text to increase the ecological validity of results.

Thus, the study was divided into three parts: In the first part, an analysis model was established according to an individual's score on the scale, and the latent traits of individuals were estimated based on IRT. In the second part, employing text mining was established to build a proactive personality classification model with essay question text and micro-blog text data. In the third part, the text mining results were used as the prior information for the IRT Bayesian estimation, and the estimation results were validated. We further compared these results with the baseline results (part 1 and part 2) to obtain the estimation results again and judge whether the prior information of the text could improve the quality of estimation.

Thus, we put forward the following research hypothesis: With the addition of prior information, the performance of the classification model will improve.

## Materials and Methods

### Dataset

#### Participants

The participants were students of Shandong Normal University, with liberal arts students and science students each accounting for 50%. First, we administered a proactive personality questionnaire and proactive personality essay questions to students, respectively, and then analyzed the scores and essays. We collected 1,671 questionnaires; among them, 901 respondents had valid micro-blog ID; respondents with invalid micro-blog ID were excluded. The sample comprised 100 boys and 801 girls: 328 freshmen, 347 sophomores, and 226 juniors. The average age of the subjects is 19.27 years (*SD* = 1.05). All the micro-blog texts of 901 subjects from their first post to January 27, 2020, were gathered by Weibo Application Programming Interfaces (APIs), resulting in a total number of micro-blog texts of 13,511 posts. After removing non-original posts (e.g., repost, advertisements, pictures, and videos), 4,955 of original posts of subjects were kept, average 5.5 posts per person. Table 1 shows the summary of the text datasets. Before the study, all participants signed an informed consent form, which was approved by the Ethics Committee of Shandong Normal University (sdnu-2017020242).

**Table 1:** The basic statistics of text length in three text datasets.

**Dataset**	**Mean**	***SD***	**Min**	**Max**
Micro-blog text (*N* = 901)	123.14	62.11	15	591
Essay question text (*N* = 901)	223.25	71.13	6	432
Micro-blog text + Essay question text (*N* = 901)	346.39	71.48	153	742

#### Definition of “Label”

By searching for and sorting out the texts related to proactive personality, we collected factors (such as participating in student unions, public welfare activities, innovation, and entrepreneurship competitions, etc.) that may reflect the proactive personality, then we invited 16 experts (most of them are college counselors and teachers of student division) to evaluate the importance of these factors by scoring them 0–9 points; we regarded the mean value of the importance of each factor as the corresponding weight. By adding all the scores for each factor, the total scores were used as criteria for measuring. Next, the scores were arranged in descending order, and the top 50% of subjects were categorized as the higher class of proactive personality, while the other subjects were categorized as lower class. In this manner, each subject was assigned a category label.

#### Research Tools

##### College Students' Proactive Personality Questionnaire

The modified Chinese version of an 11-item questionnaire by Shang and Gan (2009), originally developed by Bateman and Crant (1993), was used in this study. The scale was developed as a 7-point Likert-type scale with 1 representing “strongly disagree” and 7 representing “strongly agree.” Higher scores indicated higher levels of proactive personality. The coefficient alpha was 0.875 in this study.

##### College Students' Proactive Personality Essay Questions

After completing the questionnaire, the respondents were also asked to answer four questions that reflect their proactive personality (the following is an English translation of questions in Chinese): (1) In daily life, if circumstances constrain you from showcasing your talents, what would you deal with the problem? Please explain the reasons why you would do those things; (2) in daily life, when coping with the problem, will you accept and use the existing methods or are you willing to explore new methods; please explain your reasons; (3) when you deviate from your daily routine, your living environment changes, and the probability of making mistakes increases; thus, will you take action to change or not; please explain your reasons; and (4) what will happen in your academic and personal life in the next few years, and how are these predictions related to your future career? The subjects were required to fill in the questions according to their actual situations. The requested length of the answer to each question was approximately 60 Chinese characters.

### Method 1: Proactive Personality Measurement Based on IRT

#### IRT Item Selection

The program implemented by Python was used to estimate the difficulty and discrimination parameters of the proactive personality questionnaire of college students, and they were used as fixed parameters in Method 3. Table 2 shows the difficulty and discrimination information of 901 college students' proactive personality questionnaire results. A Bayesian estimation method based on MH-RM was adopted in the estimation of difficulty and discrimination, and a parallel sampling method was conducted, which made the model construction more efficient. Additionally, Metropolise-Hastings can calculate the high-dimensional integral to ensure the efficiency of data analysis, and Robbins-Monro can greatly improve the accuracy of estimate value (Guo, 2018). However, not all items were suitable for parameter estimation, and a certain number of items must be selected. Eleven items have 211 types of combinations when using the exhaustive search to find the optimal results, in which the 1-th, 5-th, 6-th, 8-th, 9-th, and 11-th item combination comprised the final classification (later classification) of the highest accuracy (Text classification results as a feature, IRT classification results as a feature, and two features combined to achieve the highest accuracy). To ensure that all studies comparing standards were consistent, only these six items were retained to use in the following parameter estimation, and five-fold cross-validation was used to determine the training set and validation set.

**Table 2:** The item parameters of 10 selected items.

**Item**	**α**	***β1***	***β2***	***β3***	***β4***	***β5***	***β6***
1	1.224	−5.209	−4.388	−4.024	−2.172	−1.392	4.544
5	1.452	−3.330	−2.228	−1.254	−1.702	8.476	1.868
6	1.318	−4.134	−2.621	−1.737	−3.787	5.857	1.785
8	1.724	−3.703	−3.205	−2.727	−1.993	−1.173	−2.927
9	1.905	−3.457	−3.134	−2.473	−1.607	−6.611	3.962
11	1.041	−4.602	−3.492	−2.687	−1.482	−4.083	8.010

#### Subjects' Proactive Personality Ability Parameter Estimation Based on IRT

The Bayesian estimation method is efficient in determining the ability parameters and response level of subjects and is then joined with the maximum-likelihood estimation method to obtain the required maximum value (Fox and Glas, 2001; Rupp et al., 2004). The key step of Bayesian estimation is to determine the distribution frequency of parameters; only in this manner can the accuracy of output be effectively improved. When we test subjects many times, we have an approximate estimate of their ability. This subjective estimation is the prior probability. The posterior probability is the result after sampling, which can be regarded as the result of adjusting prior probability with sampling information (Candy, 2016).

The item parameters and 901 subjects' response scores on the questionnaire were the input data, and the maximum posterior estimation was adopted to estimate the ability parameters. It is similar to the maximum-likelihood estimation, but the prior distribution of the parameters is multiplied after the likelihood function. Maximum-likelihood estimation considers that θ is the best when *P*(*X*|θ) achieves the maximum value; in this case, the maximum-likelihood estimation regards θ as a fixed value, but is an unknown value. The maximum posterior probability distribution considers that θ is a random variable and has a certain probability distribution, called a prior distribution. The prior distribution *P*(θ) should also be considered; therefore, θ is the best when *P*(*X*|θ) *P*(θ) has achieved the maximum value (Helin and Burger, 2015). Because *P*(*X*) (the prior distribution of *X*) is fixed (which can be obtained by analyzing the questionnaire), the maximization function (He, 2013; Zhang et al., 2019) can be changed into:

(4) $$\begin{array}{l} \begin{array}{l} P\left(\theta |X\right)=\;\frac{P\left(X|\theta \right)P\left(\theta \right)}{P\left(X\right)}\; \end{array} \end{array}$$

According to the Bayesian principle, *P*(θ) must be maximized; the maximum posterior probability estimation (He, 2013; Zhang et al., 2019) is,

(5) $$\begin{array}{l} \begin{array}{l} argmaxP\left(\theta |X\right)=argmax\frac{P\left(X|\theta \right)P\left(\theta \right)}{P\left(X\right)}\propto \arg maxP\left(X|\theta \right)P\left(\theta \right)\; \end{array} \end{array}$$

#### The Ability Parameters for Classification

Item Response Theory can be used to classify psychological traits by transforming the continuous variable θ into a categorical variable. The following are several IRT classification methods:

##### Logistic Regression Classification

Using the method of binary logistic regression analysis, with θ as the independent variable and the classification label as the dependent variable, the regression equation is established to predict sample category attributes and calculate the classification index information:

(6) $$\begin{array}{l} \begin{array}{l} logit\left(p\right)=\ln\left[\frac{p}{1-p}\right]=ax+b\; \end{array} \end{array}$$

When the cutoff point is 0.5, that is, when *p* > 0.5, the sample is classified as a high proactive personality; conversely, when *p* < 0.5, the sample is classified as a low proactive personality. This method was used in this study.

##### Use the Midpoint to Get the Classification Threshold

This method requires finding the median of the two distributions (high classification and low classification) and then taking the midpoint of the two medians (Cizek and Bunch, 2007). For example, the median of the distribution of the high proactive personality and the low proactive personality is 0.5 and −0.5, respectively, and the midpoint is 0, which is the classification threshold.

##### Use the Best F1 Classification Threshold

Assuming that there are *n* ability parameters, the ability parameters are ranked high and low, and the first parameter is selected as the threshold for classification. The subjects whose ability parameters are lower than this threshold will be classified as the low category and the others will be classified as the high category. In this condition, we can calculate an F1 value according to the classification result. Then select other ability parameters in turn as thresholds to calculate the corresponding F1-value, by finding the highest F1-value, the corresponding ability parameter is the best classification threshold.

**Figure 1:**
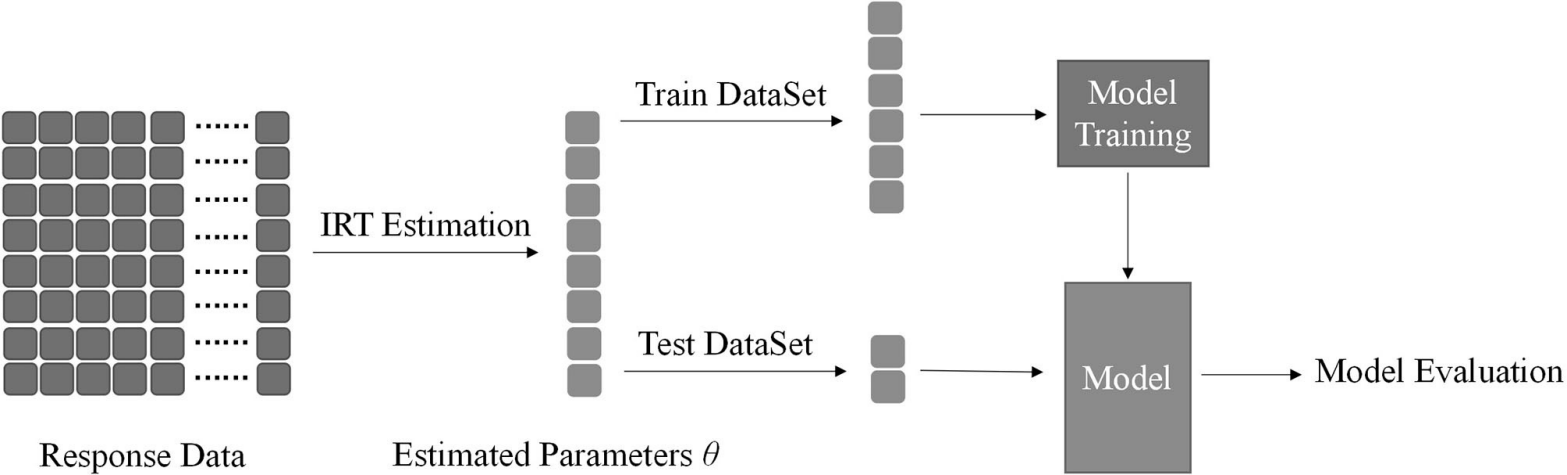
The shape of response data is *n* × *m*, where *n* is the number of subjects and *m* is the number of items. After estimation, it can return a vector of parameters θ, where its length is *n* as the same as the number of subjects. The vector is used as the input of the classification method mentioned above. Estimated parameters θ is divided into two parts called train dataset for model training and test dataset for model evaluation. We used five-fold stratified cross-validation in this process.

We divide the variable θ into two parts, which are called the train dataset and the test dataset, respectively. The latter method in the above is simple and convenient. It is a linear classifier whose input is a vector essentially. In this step, finding an appropriate threshold is a kind of model training process. It only trains on the train dataset and evaluates the test dataset. Model training processes in the method use five-fold cross-validation. Because five-fold cross-validation is adopted, the original data are divided into five parts without repeated sampling; that is, one is selected as a validation set each time, and the remaining text is selected as the training set. They account for 20 and 80% of all data, respectively, and the final results are the average of five validation sets. The following two methods also use cross-validation for model selection. The flowchart of Method 1 is shown in Figure 1.

### Method 2: Proactive Personality Measurement Based on Text Mining

#### Data Preprocessing

Essay question text part: the text of essay questions was typewritten into the computer through the iFly software (voice input software of iFlytek). All researchers who were involved were told in advance that they should follow the original intentions of subjects and cannot change the original text. Micro-blog text part: as described in the Participants part above, we have retained 4,955 original posts. The Jieba package in Python was used to perform word segmentation; next, the segmented word was compared with the stopwords lists of Harbin Institute of Technology (Guan et al., 2017), to delete pronouns, useless auxiliary words, and punctuation.

#### Feature Extraction

Term frequency-inverse document frequency (TF-IDF) was a statistical method to assess the importance of a word to a document. The importance of a word increases in proportion to the number of times it appears in a document but decreases in an inverse proportion to the number of times it appears in a corpus, that is, the frequency of a term in one document is high, and in other documents is low. Thus, we can infer that this word or phrase has a good classification ability and is suitable for classification (Huang et al., 2011). And TF-IDF is a data-driven method, not affected by word frequency. Due to the small amount of data in this study, the corresponding word frequency matrix is sparse and the corresponding thesaurus is large, the words that can represent active personality may have a small word frequency, so the TF-IDF method is adopted. The formula of TF-IDF (Wu et al., 2008) is:

(7) $$\begin{array}{l} \begin{array}{l} w\left(t,d\right)=\frac{tf\left(t,d\right)\times \log\left(\frac{N}{n_{t}}+0.01\right)}{\sqrt{\sum_{t\in d}{\left[f\left(t,d\right)\times \log\left(\frac{N}{n_{t}}+0.01\right)\right]}^{2}}}\; \end{array} \end{array}$$

In formula (7), *w*(*t, d*) denotes the weight of feature term *t, tf(t, d)* represents the word frequency of feature term *t, N* denotes the number of all training documents, *n*_*t*_ represents the number of documents with the feature term *t* in the training set, and the denominator is the normalization factor.

The essay questions text and the micro-blog text were compared to analyze the frequency of key features, and TF-IDF was used to analyze the weight of features, to facilitate the subsequent classifier training. All features will be extracted; thus, the weight of TF-IDF of all words was retained.

#### Feature Selection

This study assesses a binary classification problem, and the traditional F test was used for feature selection. The principle is that a feature's corresponding positive classification is significantly different from the negative classification, and this feature is retained. The index to measure the difference is the *F*-value and *p*-value. The larger the *F*, the smaller the *p*. The greater the difference between the positive and negative data, the better the classification task. In this study, each feature corresponds to a *p*-value, and two feature selection algorithms based on *F*-test were adopted. Retain features with *p*-value < 0.05; retain features with *p*-value < 0.1. The number of features with *p*-value < 0.01 is small, so this experiment was not conducted. Through calculation, the classification effect of retaining all features with *p*-value < 0.1 is the best; thus if the significance level is ≤ 0.1, this feature is retained; *p*-value ≤ 0.1 is the threshold value for statistical inference in the educational measures and statistics (Ovink et al., 2017). In the case of making mistakes of no more than 10%, the difference between the positive and negative data is considered significant and can be classified.

#### Classifier

Support vector machine (SVM) was used to establish the corresponding relationship between features and labels and then build the analysis model. Support vector machine is a common classification model that can complete the linear or non-linear classification of text according to certain intervals and has a significant advantage in non-linear information classification. The learning strategy of SVM is to adopt and maximize the interval method for the information classification, the equivalent of a convex quadratic program to solve, through calculating and selecting the optimization algorithm, classifying dataset efficiently and accurately (Yin and Hou, 2016).

**Figure 2:**
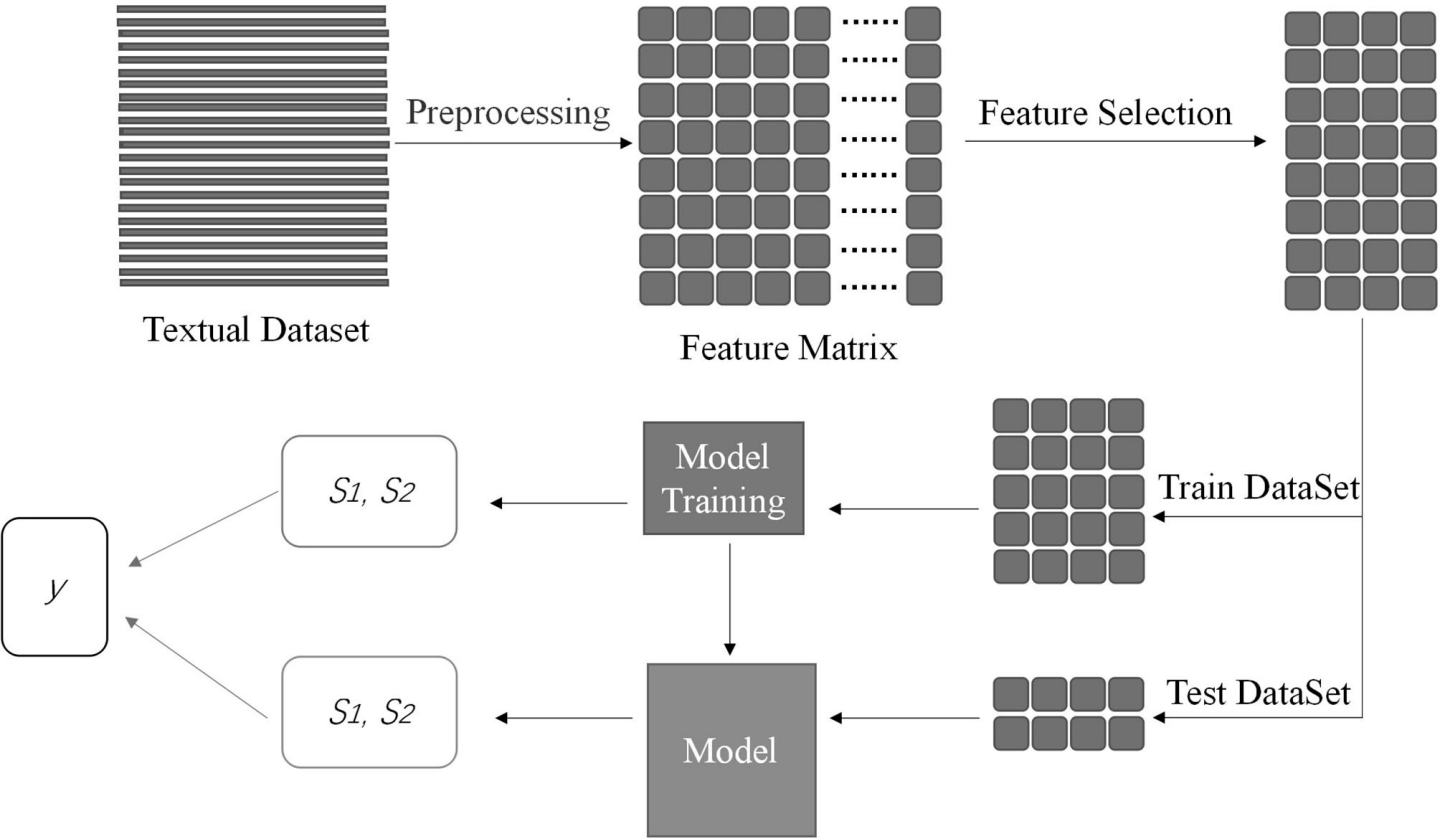
In the figure, the dense bar represents the subject's text. After the preprocessing, the textual data transforms into feature matrix with a size of *n* × *c*, which *n* is the number of the subjects and *c* is the number of the corpus. The combined block represents the matrix. Feature selection can extract useful information for classification. Then, divide the matrix into two parts, namely, train and test dataset. Other procedure is similar to Figure 1. At the end of the procedure, there are two boxes. Two boxes contain *S1* and *S2* got from train dataset and test dataset. The likelihood ratio *y* is made up of them.

After the step of feature selection, the number of columns of the textual feature matrix has been greatly reduced, where the rows of the matrix represent the subjects and the columns of the matrix represent words the weight of the corpus. Similar to Method 1, we divide the matrix into the train and test datasets for model training. When every model training finished, we recorded category probability in the train and test dataset, defining positive category probability and negative category probability as *S1* and *S2*. The likelihood ratio is defined as *y* = *ln (S1/S2)*. *y* is a vector with a size of *n*, where *n* is the number of subjects. Because five-fold cross-validation is applied in this method, we recorded *y* five times, and *y* consists of the train and test part every time. It corresponds to Method 1. The flowchart of Method 2 is shown in Figure 2.

### Method 3: Proactive Personality Measurement Based on IRT and Text Mining Combined

In Method 3, to combine the data of the text and questionnaire in the Bayesian framework, the score from text mining in Method 2 as *a priori* information was input into the distribution model in Method 1 (He et al., 2014). The likelihood ratio *y* mentioned above is used as *a priori* information for the IRT parameter estimation, and it can modify the default prior distribution of ability parameters. For example, theoretically, the default of ability parameters follows the standard normal distribution. The prior information we give through machine learning may modify the ability distribution of a subject to *N* ~ *(0.05, 1.4)*.

Under the Bayesian framework, the product of prior distribution and likelihood ratio is proportional to the posterior distribution of traits. The corresponding formula (He et al., 2014) is:

(8) $$\begin{array}{l} \begin{array}{l} P\left(\theta |x,y\right)\propto P\left(x|\theta ,\alpha ,\beta \right)g\left(\theta |y\right) \end{array} \end{array}$$

Through the analysis of the above formula, *x* represents the answer matrix of the questionnaire, *y* represents the score of the text, *g*(θ|*y*) denotes the prior information of the text, and α and β represent the difficulty level and discrimination of the entire project. *P*(*x*|θ, α, β) denotes the likelihood equation of the model. According to the above formula, we can build the following linear regression model, then:

(9) $$\begin{array}{l} \begin{array}{l} y_{n}=b_{0}+b_{1}\theta_{n}+\varepsilon_{n} \end{array} \end{array}$$

Through the analysis of the above formula, *b*_0_ and *b*_1_, respectively, represent the regression coefficient and intercept of the equation, assuming that the total number of tests is *N* and conforms to the normal distribution, then when the type of text is fixed, θ_*n*_ satisfies the normal distribution of the equation:

(10) $$\begin{array}{l} \begin{array}{l} \theta_{n}|y_{n}∽N\left(b_{0}+b_{1}y_{n},\sigma^{2}\right) \end{array} \end{array}$$

The above formula can be used to represent the prior distribution of inherent traits of proactive personality. After obtaining the potential trait parameters, two features are constructed together with the prior information to classify.

**Figure 3:**
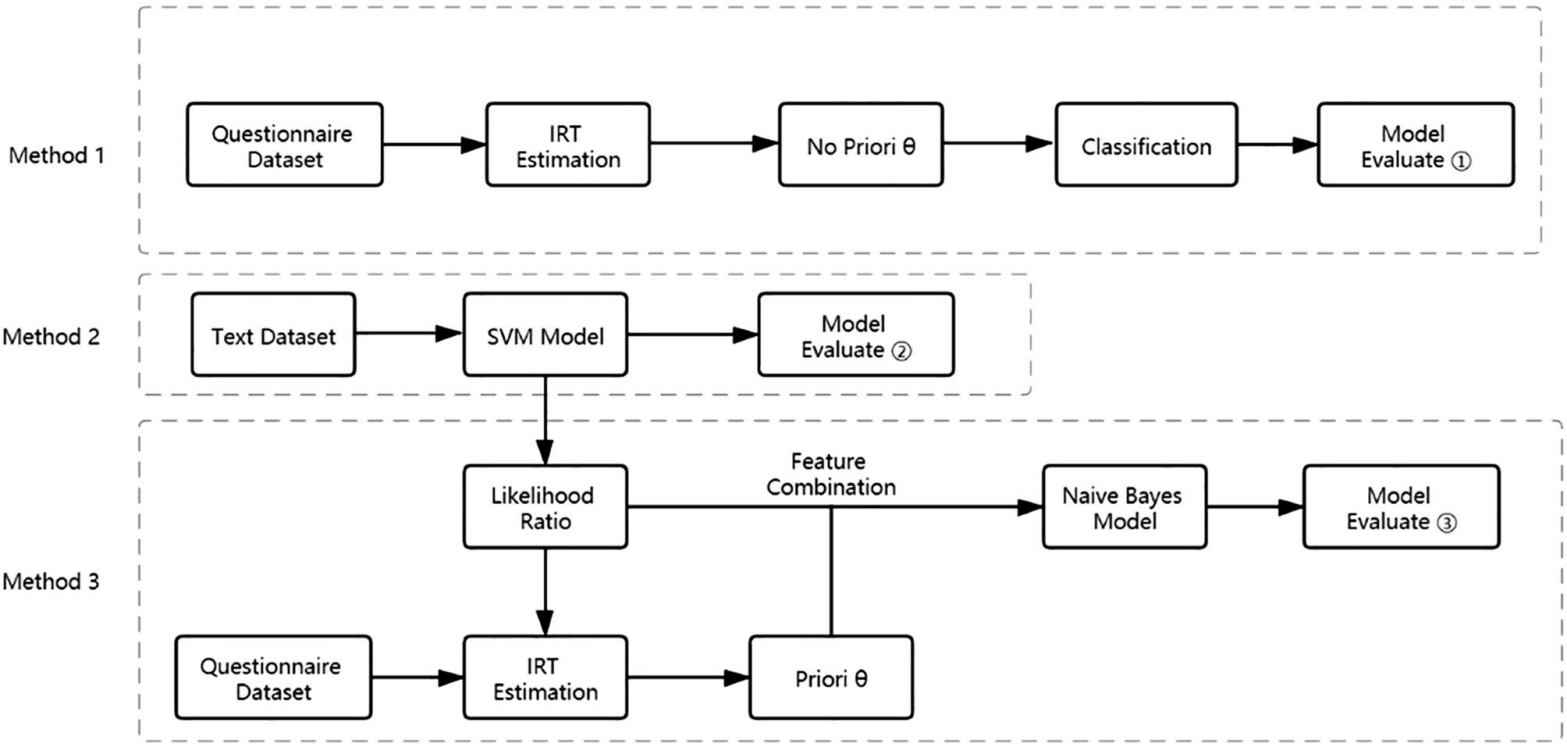
The flowchart of this study.

When estimating the latent trait of subjects, the MH-RM method can be adopted, and Python software is a helpful tool to achieve the parameter estimation. To verify whether the estimation accuracy will be improved when the prior information is introduced, the ability parameter can be added from Methods 1 and 2. Next, the results of Methods 1 and 2 were compared with the results of Method 3, which were obtained by Bayesian estimation to complete the verification. The relationship between the three methods is shown in Figure 3.

**Figure 4:**
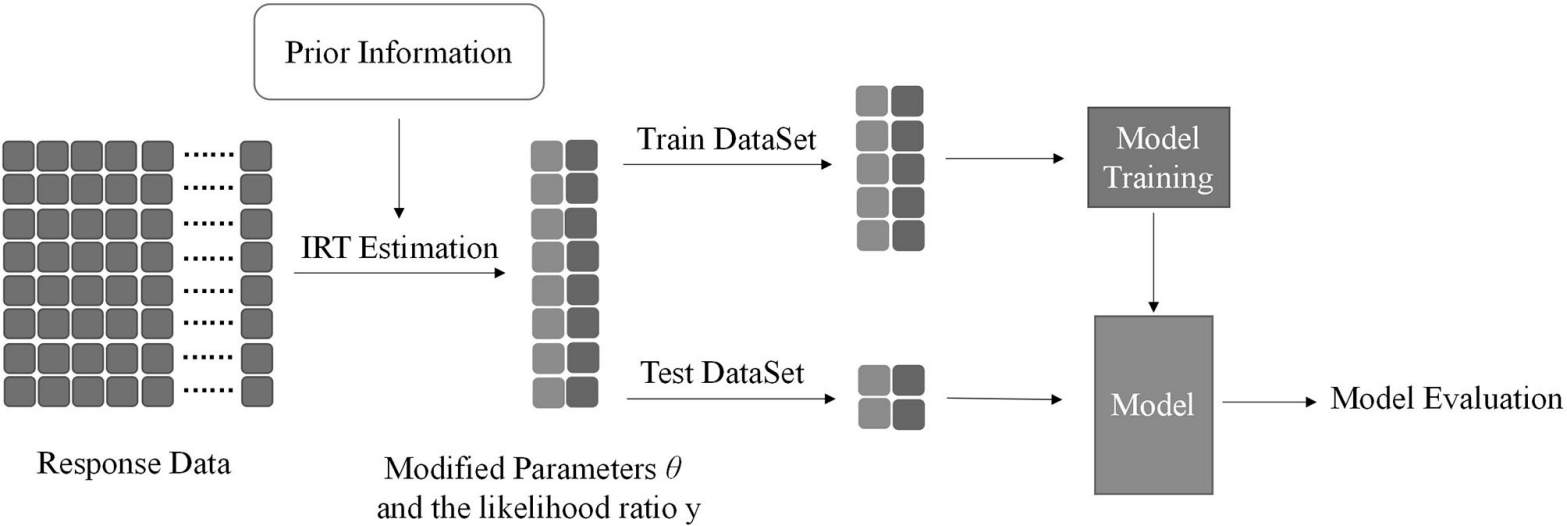
Similar to Figure 1, the basic procedure is coincident. Prior information obtains from the likelihood ratio *y*. Then, concatenate θ and *y* together. We won't repeat the rest of the procedure here.

We get modified parameters θ after the above process. The likelihood ratio *y* is the compression of classification information in machine learning. When *y*_*n*_ is more than 0, it is predicted to be a positive category, and when *y*_*n*_ is < 0, it is predicted to be a negative category. We have three types of text datasets (essay question text, micro-blog text, and combination of both), and select the *y* corresponding to the test dataset with the best comprehensive evaluation index as the prior information for parameter estimation to obtain θ. Concatenate *y* and θ into a matrix of size *n* × *2*, and then select the naive Bayes classifier for model training. Similarly, cross-validation is used for model selection. The flowchart of Method 3 is shown in Figure 4.

## Results

The three methods generated seven models, including one IRT classification model, three text classification models, and three combined models. Six evaluation indicators [accuracy, sensitivity (recall), specificity, positive predictive value (precision), negative predictive value, and F1 score (F1)] were used to evaluate the classification effect of the models. The F1 is interpreted as a weighted average of the precision and recall, where F1 score is also termed *F* measure. When the precision and recall indicators are contradictory, F1 is used as an indicator for comprehensive consideration. There calculation methods of indicators are as follows (Table 3):

(11) $$\begin{array}{l} \begin{array}{l} Accuracy=\frac{a+d}{a+c+b+d}\; \end{array} \end{array}$$

(12) $$\begin{array}{l} \begin{array}{l} Sensitivity=\frac{a}{a+c}\; \end{array} \end{array}$$

(13) $$\begin{array}{l} \begin{array}{l} Specificity=\frac{d}{b+d}\; \end{array} \end{array}$$

(14) $$\begin{array}{l} \begin{array}{l} Positive\;Predictive\;Value=\frac{a}{a+b}\; \end{array} \end{array}$$

(15) $$\begin{array}{l} \begin{array}{l} Negative\;Predictive\;Value=\frac{d}{c+d} \end{array} \end{array}$$

(16) $$\begin{array}{l} \begin{array}{l} F1=\frac{2}{\frac{1}{sensitivity}+\frac{1}{positive\;predictive\;value}} \end{array} \end{array}$$

**Table 3:** 2 × 2 confusion matrix table.

	**True Standard**	
	**C_**1**_**	**C_**2**_**
Assigned C_1_	a	b
Assigned C_2_	c	c

### The Results of Method 1 and Method 2

#### Ability Parameter Estimation Results

In Table 4, the proactive personality of college students under the IRT method is within the range of [−3.998, 1.962], and the average value is approximately 0.001.

**Table 4:** The summary of θ parameters in IRT classification.

**Classification**	***Min***	***Max***	***Mean***	***SD***
IRT	−3.998	1.962	0.001	0.875

#### IRT or Text Classification Results

In Table 5, for the important indicator to evaluate the text classification, the accuracy of the IRT classification is 0.602, and the accuracy of the three text classification methods (essay question text, micro-blog text, and essay question text + micro-blog text) are 0.802, 0.751, and 0.823. This result shows that the result of IRT classification alone achieves the lowest accuracy among the four methods. High sensitivity means that individuals with high proactive personalities can be well-identified, but the sensitivity of the four models is not high, and the highest is only 0.674 (essay question text + micro-blog text). The specificity of the essay question text model is the highest, reaching 0.959. Positive predictive value and negative predictive value can both reflect the confidence degree of correct classification. As for predictive value, the higher the predictive value, the more reliable the result; besides IRT classification, the positive and negative predictive values of the three text classification models were higher than 0.8 and 0.7, respectively. F1 considers sensitivity and positive predictive value comprehensively. The above four models are all within the acceptable range; the highest is 0.778 (essay question text + micro-blog text).

**Table 5:** The results of the four models in IRT classification and text classification.

**Classification**	**ACC**	**SEN**	**SPE**	**PPV**	**NPV**	**F1**
IRT	0.602	0.626	0.577	0.611	0.604	0.613
Essay Question Text	0.802	0.614	0.959	0.927	0.746	0.739
Micro-blog Text	0.751	0.578	0.897	0.827	0.715	0.681
Micro-blog Text + Essay Question Text	0.823	0.674	0.948	0.918	0.775	0.778

### The Results of Method 3

#### Ability Parameter Estimation Results

In Table 6, the ability parameters of proactive personality of college students are both within the range of [−3.999, 3.643] under the combination of the three text classifications and IRT, with an average value of approximately 0.040.

**Table 6:** The summary of θ parameters in text–IRT classification.

**Classification**	**Min**	**Max**	**Mean**	**SD**
Essay question text + IRT	−3.999	3.643	0.049	1.168
Micro-blog text + IRT	−3.999	3.642	0.036	1.167
Essay question text + Micro-blog text + IRT	−3.999	3.512	0.049	1.164

#### Text–IRT Combination Classification Results

In Table 7, when prior information is added, the classification accuracy of the three methods + IRT (essay question text + IRT, micro-blog text + IRT, and essay question text + micro-blog text + IRT) are 0.731, 0.693, and 0.849, respectively, which are higher than that of IRT classification, indicating that the accuracy is improved after prior probability is added. The combination of micro-blog text, essay question text, and IRT can achieve the highest accuracy of 0.849. The most sensitive is essay question text + IRT, reaching 0.821. The specificity of the essay question text is the highest, reaching 0.959, and shows that it is best to identify a low proactive personality. The positive and negative predictive values of several methods of text + IRT are all higher than 0.65. Among them, the positive and negative predictive values of the essay question on the text + micro-blog text + IRT method are as high as 0.879 and 0.822, respectively. The F1 of the essay question on the text + micro-blog text + IRT method is the highest, reaching 0.844.

**Table 7:** The results of the three models in text–IRT classification.

**Classification**	**ACC**	**SEN**	**SPE**	**PPV**	**NPV**	**F1**
Essay Question Text + IRT	0.731	0.821	0.648	0.676	0.802	0.742
Micro-blog Text + IRT	0.693	0.677	0.711	0.731	0.655	0.703
Micro-blog Text + Essay Question Text + IRT	0.849	0.811	0.887	0.879	0.822	0.844

## Discussion

The present study aims to propose a novel method of assessing proactive personality among college students to offer a more accurate measurement of proactive personality by combining text mining and IRT, with subjective and objective data. When employing this novel method, the essay question text and the original Sina micro-blog text are used as a subjective part; the ability parameters of the questionnaire calculated from IRT are used as the objective part. Three methods were conducted in the study. Method 1 and Method 2 developed proactive personality models based on IRT and text mining, respectively. Method 3 utilized the text mining results as the prior information for the IRT estimation to build a proactive personality evaluation model.

The results of the three methods were analyzed and compared based on six indicators (accuracy, sensitivity, specificity, positive predictive value, negative predictive value, and F1-score) to verify the validity of classification results, to find out whether the introduction of prior information would improve the performance of model prediction.

### Comparison of the Four Proactive Personality Classification Models Based on IRT and Text Classification, Respectively

As the main intent of this study was to verify if adding prior information can improve the classification performance, Method 1 performed a basic IRT estimation without adding prior information, and we got the following results: The ability traits of college students were within the range of [−3.998, 1.962], the average was approximately 0.001, and no large fluctuation was observed.

The IRT classification method was based on the traditional questionnaire, and its accuracy was 0.602. Simultaneously, the three models of text classification (essay question text, micro-blog text, and essay question + micro-blog text) were all higher than 0.750, which was significantly higher than the IRT classification. When measuring the psychological state of students, the relevant texts such as diaries, memos, and daily records of students can also provide valuable information (He, 2013; Zhang et al., 2019). He and Veldkamp (2017) used life stories of college students for text classification to assess personality adaption, and the text classification resulted in over 70% accuracy compared with the accuracy of the human-raters' results. We found that text classification was also better than IRT classification in other indicators, and it is explained in detail in Section Performance comparison of seven models based on three methods of the Discussion.

### Comparison of the Three Proactive Personality Classification Models Based on the Text–IRT Combination

Method 3 employed a novel way to assess proactive personality which combined text mining and IRT to evaluate the individual psychological state from subjective and objective aspects. The text features were used as the prior information, and the parameters obtained from IRT were used as the input, and naive Bayes was adopted to complete the test of classification results. The ability parameter of Method 3 was within the range of [−3.999, 3.643], and the average value was constant at approximately 0.040. The validity of the novel method mainly refers to the validity of classification. Generally, the validity coefficient >0.4 means that the data consistency is high (Wang and Sun, 2011), whereas the correlation between the proactive personality and the new classification standard in this study was 0.423; thus, it fulfills the requirements.

In the comparison of three text–IRT combination models, the classification accuracy is 0.731, 0.693, and 0.849, respectively. The model training effect of combining both the essay question text and micro-blog text as *a priori* information is better than those of them separately. Thus, various text information can improve the accuracy of classification. Notably, the accuracy of the two methods that include essay question text was mostly higher than that of the method without essay question text. He et al. (2012) found that text classification using a patient's self-narrative has high accuracy, which showed the importance of subjective texts to reveal psychologic traits. In the comparison of other indicators of the three combination methods, the essay question text + IRT model reached the highest sensitivity, which means that text as *a priori* information had advantages in identifying individuals with high proactive personalities. Essay question text + micro-blog text + IRT model reached the highest specificity and could be used to identify low proactive personality. In addition, the essay question text + micro-blog text + IRT model reached the highest F1, which indicated that the model had the best comprehensive performance.

### Performance Comparison of Seven Models Based on Three Methods

In terms of accuracy, the three models of text classification (essay question text, micro-blog text, essay question + micro-blog text) used as a baseline were higher than 0.750, and IRT estimation alone had the lowest accuracy of 0.602. Thus, the effect of text classification is obviously better than that of IRT classification, which may be caused by that the limited number of items of the questionnaire, which might not be comprehensive enough to fully reflect the proactive personality of individuals. But when the two kinds of texts were combined with IRT, the accuracy had been significantly improved to 0.849, which was the highest among those seven models. We could infer that the addition of free expressed text provides more information and improves accuracy.

As for sensitivity, this result showed that IRT classification and text classification had similar effects in identifying individuals with high proactive personalities. However, neither IRT classification nor text classification had better sensitivity than the three combined methods. In particular, the sensitivity of the essay question text + IRT model reached 0.821, which showed that when the text mining results as prior information was added to the IRT classification model, the sensitivity could be significantly improved.

From the perspective of specificity, the three text classification methods reached a minimum score of 0.897. Compared with the IRT classification and the combined method, their advantages were obvious. Thus, text information may be a better material to reflect a low proactive personality.

The combined method of using essay question text, micro-blog text, and IRT data had the highest F1-value, reaching 0.844, and the F1 of other methods was between [0.681, 0.771]. Through results shown in Tables 5, 7, we can infer that when prior information is added or data sources (essay question text, micro-blog text, and IRT data) become diverse, the F1 of the model will increase, but the magnitude of the increasing needs to be further studied. Diverse data sources and adding prior information may provide more comprehensive information about the proactive personality of individuals; thus, the comprehensive index F1 will increase.

Since the IRT classification was based on the questionnaire data, the design of the questionnaire or the estimation of the IRT parameters may cause some deviation between the true θ and the estimated θ. In addition, the parameters used for classification in IRT estimation were one-dimensional features, so the information provided by IRT estimation may be insufficient. To compare with, text classification had numerous features, which may be 10 times or even hundreds of times than that of IRT. Obviously, the more features utilized, the more accurate results could be obtained. According to the evaluation indicators, the freely expressed text could be used alone to assess proactive personality, and the text classification had better performance than IRT classification. What's more, freely expressed text can complement some deficiencies of IRT data under some conditions, for example, 7-point Likert scale reflects the state of the participants, unstructured freely expressed text (essay question text, micro-blog text) can provide valuable information and it will not be restricted by the options of the questionnaire, which enables the participants to fully express their inner thoughts, reflecting their proactive personality from many aspects. Additionally, the free text has higher ecological validity than questionnaire measurement.

In summary, utilizing the free texts as the prior information for the IRT estimation could reduce the deviation of IRT classification and the method of combining text classification and IRT had better performances in terms of accuracy, sensitivity, and F1, which was in line with the hypothesis put forward in the beginning. Text classification, IRT classification, and the combined methods had different and respective advantages in sensitivity, specificity, accuracy, and F1. Thus, in future studies, we should be aware that research model selection should be based on actual needs (Fabio and Maree, 2012). For instance, if accuracy is the major pursuit, then the text–IRT method is your suitable choice, under such circumstances, sensitivity and specificity may be sacrificed to some degree.

## Limitations and Further Work

In terms of data collection, since the ratio of gender in Shandong Normal University is 3–7, the sample used was not gender-balanced. It is worth mentioning that China's research on gender differences in the proactive personality of college students is inconsistent (Huang and Liu, 2011; Wu et al., 2016); it may lead to an imbalance prediction.

In designing essay questions, the four macro-level questions left too much room for the participants. In future research, we can further refine the questions and link them with the actual situation to evaluate traits from a more micro-level perspective (Yang and Chau, 2016). In addition, more related resources that reflect proactive personality can be added to the labels (Morgeson et al., 2012; Wu and Griffin, 2012).

Refer to the relationship between proactive personality and work outcomes; Li et al. (2014) showed that proactive personality can change, and the direction of causality could go in both ways. This was a limitation of both our research and prior research on proactive personality.

## Conclusions

The present study combined text mining and IRT to offer a new combination method for measuring proactive personality, which has not been done by previous research. The research findings were properly supported by validity and reliability. Thus, we believe the approach proposed by this study may be currently the best performed evaluation model of proactive personality. The text classification method generally had higher accuracy and the IRT classification method had lower accuracy, but the accuracy was improved after adding the prior information of the free text, and the method of combining essay question text, micro-blog text, and IRT had the highest accuracy. If sensitivity is emphasized and participants with low proactive personality need to be correctly identified, the text + IRT method was better, and the essay question text + IRT method had the highest sensitivity. In terms of specificity, the IRT classification method had a poor effect, text classification was the best method to identify participants with high proactive personalities, and the text + IRT method as also above the medium level. If the model was considered comprehensively, the method of essay question text + micro-blog text + IRT with the highest F1 score could be selected.

Proactive personality is an important predicting variable in the career development of individuals. The era of big data psychology provides a novel method for measuring proactive personality. Through adjustments and adaptations, companies or career guidance departments can better evaluate the proactive personality of candidates and select appropriate candidates. What's more, this method could guide the planning and implementation of measuring other psychological traits, which may be progress in psychometrics.

## Data Availability Statement

The raw data supporting the conclusions of this article will be made available by the authors, without undue reservation.

## Ethics Statement

The studies involving human participants were reviewed and approved by the Ethics Committee of Shandong Normal University. The patients/participants provided their written informed consent to participate in this study.

## Author Contributions

GZ involved in conceptualization, methodology, formal analysis, software, visualization, writing the review, and editing. YZ and JW involved in conceptualization, methodology, funding acquisition, writing the review, and editing. FZ involved in conceptualization, methodology, visualization, software, writing the review and editing. XZ, MW, and YS involved in methodology, visualization, writing the review and editing, and funding acquisition. PW involved in conceptualization, methodology, and funding acquisition. All authors contributed to the article, and approved the submitted version.

## Conflict of Interest

The authors declare that the research was conducted in the absence of any commercial or financial relationships that could be construed as a potential conflict of interest.
